# Supplementary figures and images for: Intraspecific diversity loss in a predator species alters prey community structure and ecosystem functions
Source: PLoS Biol. 2021 Mar 11;19(3):e3001145. doi: 10.1371/journal.pbio.3001145 (PMC7987174; doi:10.1371/journal.pbio.3001145)

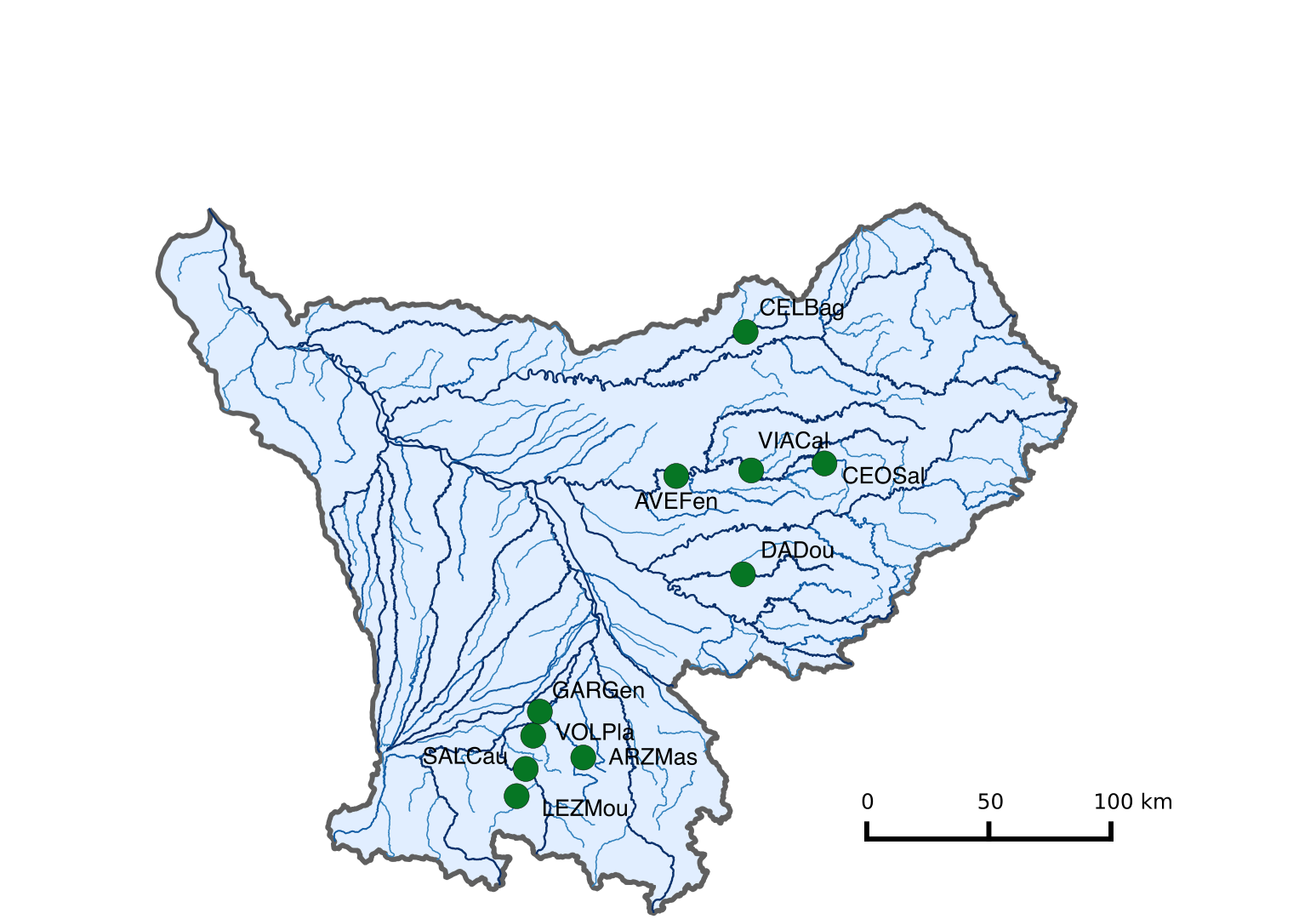

Supplement: S2 Fig — This map was drawn based on data from http://www.geoinformations.developpement-durable.gouv.fr/bd-carthage-r363.html. (PNG) [file pbio.3001145.s002.png]

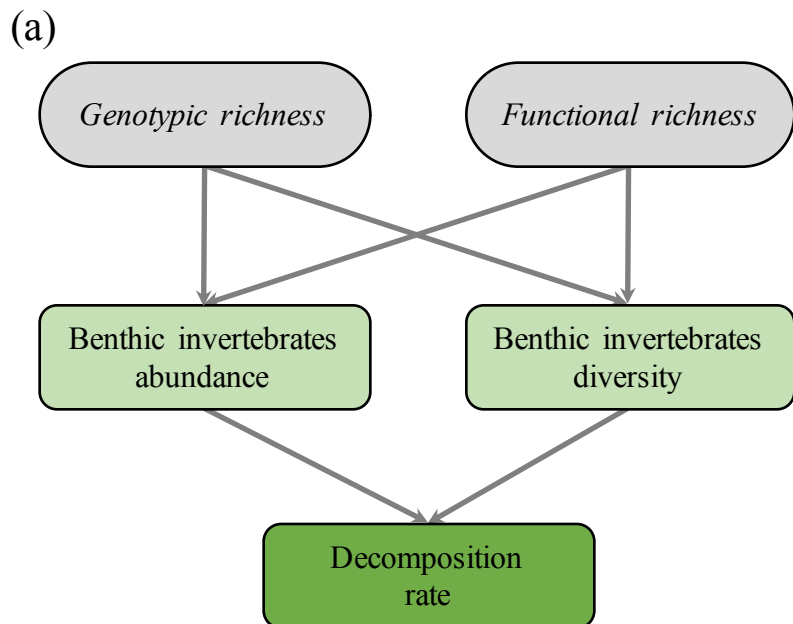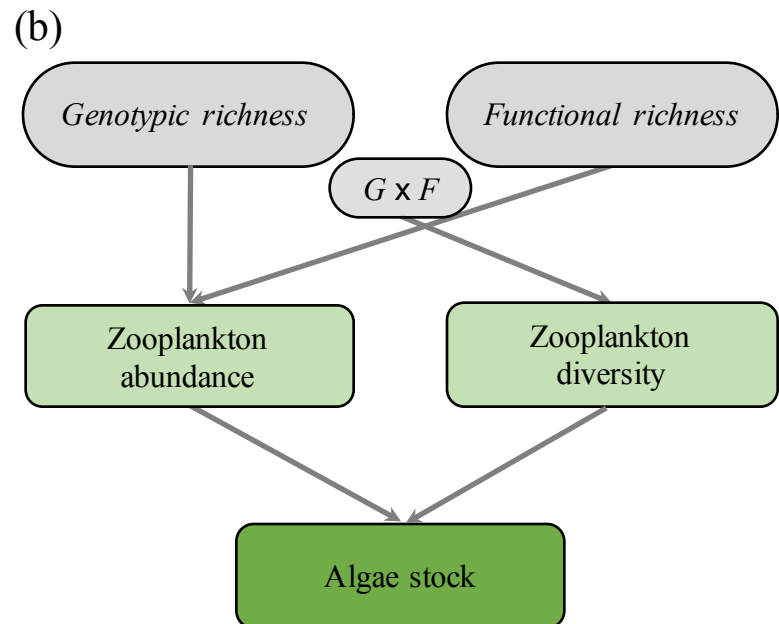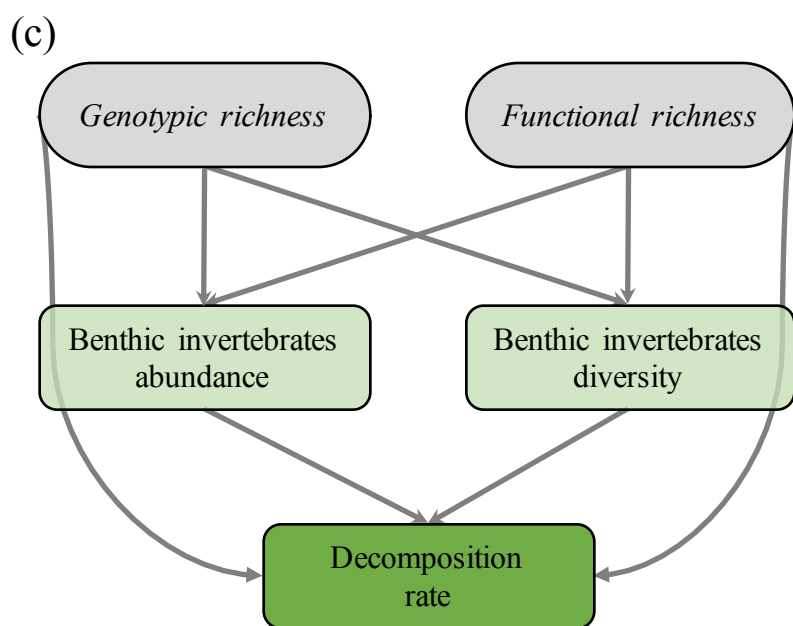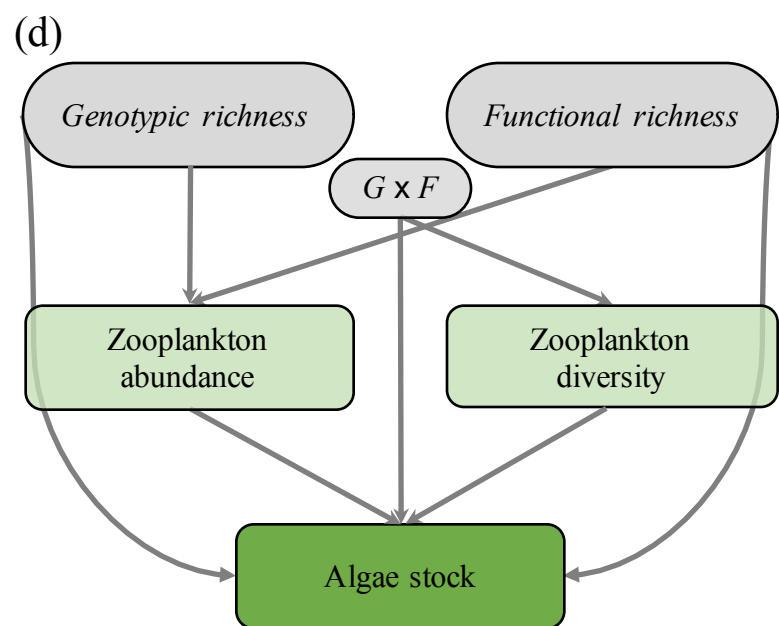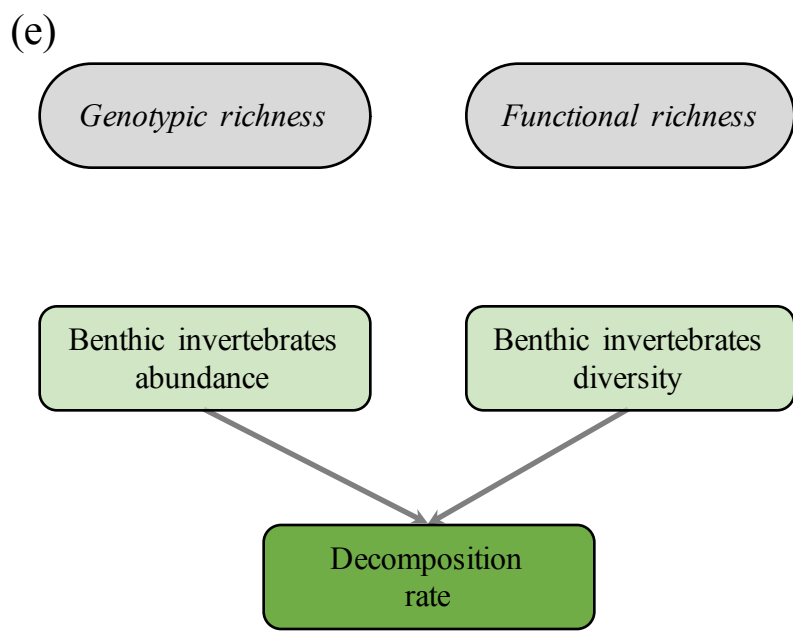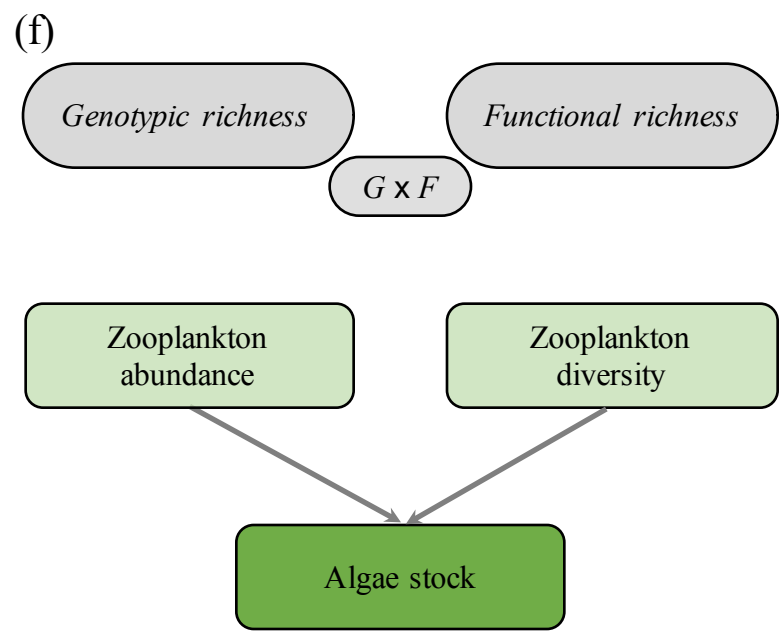

Supplement: S5 Fig — Diagram of the causal pathways used to explain variation in (a) decomposition rate and (b) algae stock. These models were compared to alternative models ((c) and (d), respectively) including direct effects of genotypic and functional richness on ecosystem functions. Finally, simplified models were performed, in which the effects of genotypic and functional richness on community structure were excluded ((e) and (f)). “G x F” denotes the interaction between genotypic and functional richness. (PDF) [file pbio.3001145.s005.pdf]
