# Supplementary material for: Intraspecific diversity loss in a predator species alters prey community structure and ecosystem functions
Source: PLoS Biol. 2021 Mar 11;19(3):e3001145. doi: 10.1371/journal.pbio.3001145 (PMC7987174; doi:10.1371/journal.pbio.3001145)
Supplement: S4 Fig — The higher the distance, the more influential the points on the variable. The horizontal bar, representing the mean across all tanks multiplied by 4, is given as an indicative threshold above which a point may be considered as influential. Tank 13 was influential in all variable and was therefore discarded from analyses. The data underlying this Figure can be found at https://doi.org/10.6084/m9.figshare.12459065.v7. (PDF) [file pbio.3001145.s004.pdf]

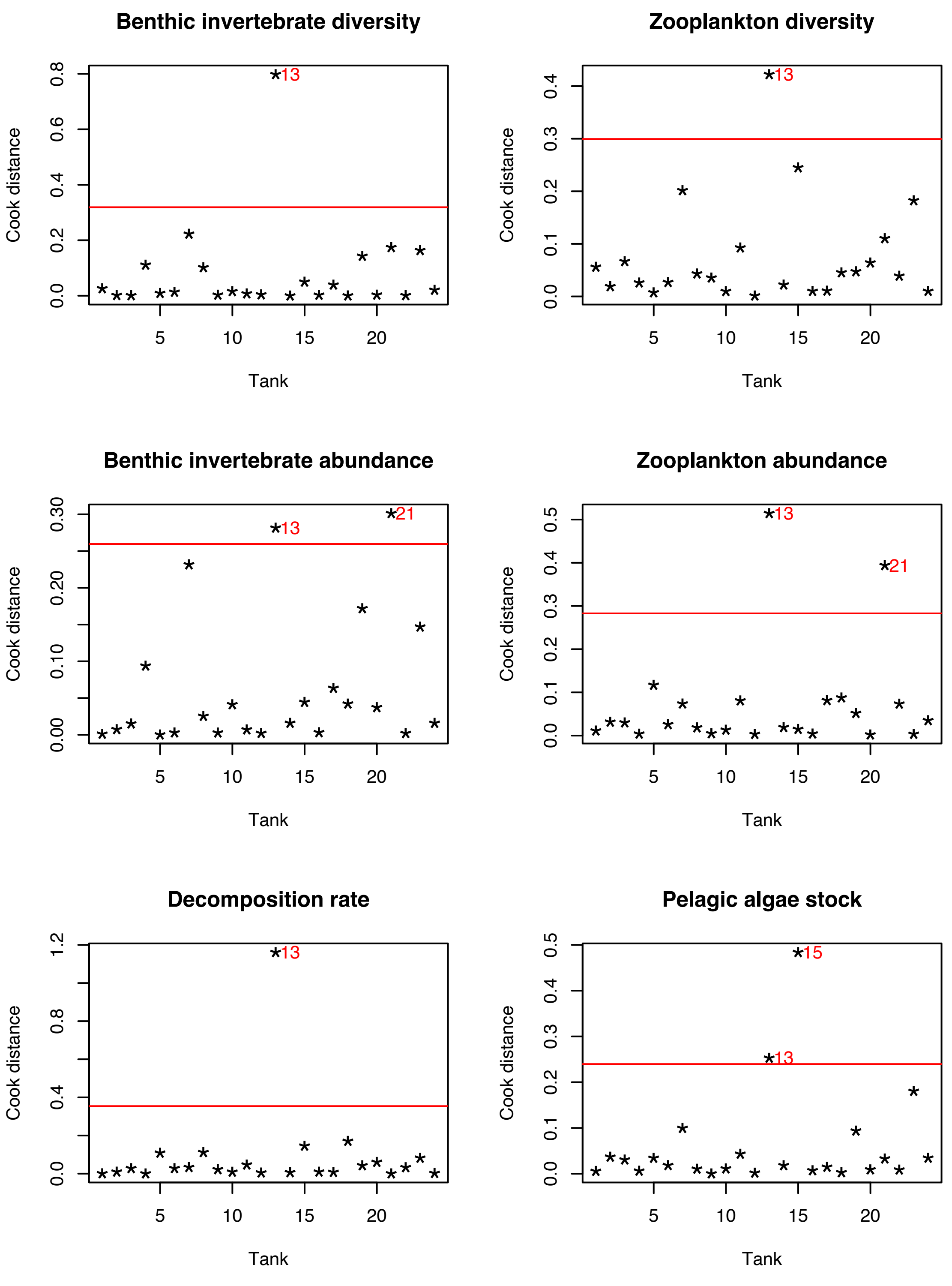

Benthic invertebrate diversity
Zooplankton diversity
Benthic invertebrate abundance
Zooplankton abundance
Decomposition rate
Pelagic algae stock
Cook distance
Tank
13
21
15
